# Muscle Regeneration Failure May Lead to Clinical Features of Myopathy in Anti-IgLON5 Disease

**DOI:** 10.1212/NXI.0000000000200525

**Published:** 2026-07-13

**Authors:** Yvette S. Crijnen, Verena Endmayr, Suzanne C. Franken, Nadine A.M.E. Van Der Beek, Maartje Louter, Thomas Ströbel, Ellen Gelpi, Inga Koneczny, Ryuichi Shigemoto, Jan Lewerenz, Birgit Högl, Julia V. Wanschitz, Ulrich He Hofstadt-Van Oy, Robin W. Van Steenhoven, Mariska M.P. Nagtzaam, Jeroen Kerstens, Juliette Brenner, Anna E.M. Bastiaansen, Robert M. Verdijk, Thierry P.P. Van Den Bosch, Marco Schreurs, Sharon Veenbergen, Marcel M. Verbeek, Dave L. Roelen, Mar Guasp, Peter A.E. Sillevis Smitt, Lidia Sabater, Juna M. de Vries, Romana Höftberger, Maarten J. Titulaer

**Affiliations:** 1 Department of Neurology, Erasmus University Medical Center, Rotterdam, the Netherlands;; 2 Division of Neuropathology and Neurochemistry, Department of Neurology, Medical University of Vienna, Austria;; 3 Comprehensive Center for Clinical Neurosciences and Mental Health, Medical University of Vienna, Austria;; 4 Department of Clinical Neurophysiology, Erasmus University Medical Center, Rotterdam, the Netherlands;; 5 Institute of Science and Technology Austria (ISTA), Klosterneuburg, Austria;; 6 Department of Neurology, Ulm University, Germany;; 7 Department of Neurology, Medical University of Innsbruck, Austria;; 8 Department of Neurology, Knappschaft-Kliniken Dortmund, Germany;; 9 Department of Pathology, Erasmus University Medical Center, Rotterdam, the Netherlands;; 10 Department of Immunology, Erasmus University Medical Center, Rotterdam, the Netherlands;; 11 Department of Neurology, Radboud University Medical Centre, Donders Institute for Brain, Cognition and Behaviour, Radboud Alzheimer Centre, the Netherlands;; 12 Department of Human Genetics, Radboud University Medical Centre, Nijmegen, the Netherlands;; 13 Department of Immunology, Leiden University Medical Centre, the Netherlands;; 14 Department of Neurology, University of Barcelona, Spain;; 15 Neuroimmunology Program, Fundació de Recerca Clínic Barcelona-Institut d´Investigacions Biomèdiques August Pi I Sunyer-Caixa Research Institute, Universitat de Barcelona, Spain; and; 16 Centre for Biomedical Network Research on Rare Diseases (CIBERER), Instituto de Salud Carlos III, Madrid, Spain.

## Abstract

**Background and Objectives:**

Anti-immunoglobulin-like cell adhesion molecule 5 (IgLON5) disease is a novel and potentially treatable entity. Therefore, it is important to recognize all clinical symptoms and diagnostic clues. We specifically investigated neuromuscular signs and symptoms and muscle biopsy pathology, providing a link between IgLON5 and clinical features of myopathy.

**Methods:**

All patients diagnosed with anti-IgLON5 disease in the Netherlands between 2016 and 2023 were included. Serum and CSF samples were tested with immunohistochemistry on rat brain and in-house cell-based assay using live cells. Biopsies of the vastus lateralis muscle were performed in patients with neuromuscular signs and symptoms and analyzed in Vienna together with 3 biopsies of non-Dutch patients sent to Vienna for second opinion.

**Results:**

Twenty patients with anti-IgLON5 disease were included (10 male, 50%). The median age at onset was 61.5 years (range 45–85), and the median time from onset to diagnosis was 30 months (range 3–280). Neuromuscular symptoms were present in over half of the patients (11/20), including proximal limb weakness (n = 11), axial weakness (n = 1), muscle atrophy (n = 6), and fasciculations (n = 5). All 12 muscle biopsies (9 from the Dutch cohort, 3 external) showed mild myopathic alterations, 2 additionally presented target fibers and fiber type grouping (compatible with neurogenic myopathy), and 3 patients showed immune cell infiltration. We found a strong upregulation of IgLON5 expression in muscle fibers in all patients and also in different muscle disease controls, while immunoreactivity in healthy control muscle was faint/absent.

**Discussion:**

Our data support that IgLON5 might play a role in muscle regeneration, which might result in proximal myopathy as a prominent clinical feature in anti-IgLON5 disease. This finding broadens the clinical phenotype of anti-IgLON5 disease and can be an important clue for earlier diagnosis and start of immunotherapy.

## Introduction

Anti-immunoglobulin-like cell adhesion molecule 5 (IgLON5) disease is a progressive neurologic disorder with aspects of both autoimmunity and neurodegeneration,^1^ that was first reported in 2014.^2^ Only just over 170 cases with anti-IgLON5 disease have been published since. The initial patients all had a sleep disorder, including obstructive sleep apnea, and both REM and non-REM parasomnias. In addition, bulbar symptoms, gait imbalance, and chorea were described.^2^ In recent years, the clinical phenotype of this disease has broadened, including other movement disorders and cognitive symptoms.^1^ Although neuromuscular symptoms have been mentioned in IgLON5 patients,^1^ it was unclear whether these symptoms occurred coincidentally, were an adverse effect of prescribed medications (such as steroids), or were actually associated with anti-IgLON5 disease. Previous reports suggest that early immunotherapy can be effective.^3,4^ Consequently, to improve its early recognition and treatment, a better understanding of the features of anti-IgLON5 disease is required.

The unfamiliarity of many clinicians with its wide variety of symptoms and its gradual disease course (as opposed to most other types of autoimmune encephalitis)^4^ make diagnosing anti-IgLON5 disease a challenge, which is reflected by the significant diagnostic delay. Presumably, only a small proportion of the patients with anti-IgLON5 disease is currently diagnosed.^5-7^

In this study, we aim to gain more insight into clinical symptoms and clues for diagnosis of anti-IgLON5 disease, with special emphasis on neuromuscular symptoms and muscle biopsies. In this study, we provide evidence that IgLON5 plays an important role in regeneration in human muscle pathology and its disruption might contribute to the phenotype of anti-IgLON5 disease.

## Methods

### Patients

All patients diagnosed with anti-IgLON5 disease in the Netherlands between September 2016 and April 2023 were included. National coverage was ascertained as the Erasmus MC is the academic center for neuroinflammatory disorders, the national referral center for antibody diagnostics and treatment, accredited as European Reference Network site (ERN-RITA) and the only center in the Netherlands providing anti-IgLON5 testing.

All patients alive at the time of diagnosis were seen at Erasmus MC. Patient characteristics and clinical data were obtained from detailed interviews with patients and their relatives during these visits. Thorough neurologic examination was performed. All but one patient have been treated and followed up at Erasmus MC.

### Antibody Detection and IgLON5-Specific IgG Subclasses

Serum and CSF samples were tested by immunohistochemistry (IHC) on rat brain^8^ and an in-house live cell-based assay (CBA, eMethods 1). In addition, IgLON5-specific IgG subclasses were determined using in-house CBA. In summary, Chinese hamster ovarian (CHO) cells transfected with IgLON5-expressing plasmids were incubated with the diluted patient sera (whole IgG: 1:40; IgG subclass titrations: fourfold step interval starting at 1:10 until no longer visibly positive) or CSF (1:4) for 30 minutes at 37°C.

### Tissue Analyses

Muscle biopsies of the vastus lateralis muscle were performed in 9 Dutch patients with neuromuscular signs and symptoms (cohort A). They were sent to the Department of Pathology of Erasmus MC and were forwarded to the Medical University of Vienna for second opinion. In addition, 3 muscle biopsies and one sural nerve sample of 3 non-Dutch patients with anti-IgLON5 disease (including one deceased patient in whom the biopsy was obtained at autopsy) sent to Vienna for regular care or second opinion between 2015 and 2021 were analyzed in parallel (cohort B).

Snap-frozen muscle tissue sections were investigated with routine histochemical stains including hematoxylin and eosin (H&E), myofibrillar adenosine triphosphatase (ATPase), nicotinamide adenine dinucleotide (NADH), succinate dehydrogenase combined with cytochrome c oxidase (SDH/COX), modified Gomori trichrome, periodic acid-Schiff (PAS), and Oil Red O. Immunohistochemistry was performed on sections of snap-frozen muscle tissue using antibodies listed in eMethods 2. Ultrathin sections were prepared for electron microscopy and examined with transmission electron microscopy. Western blots were performed following standard protocols. The membranes were incubated with the polyclonal rabbit primary antibody anti-IgLON5 that targets amino acids 218–289 of human IgLON5. For both Western blot and immunohistochemistry, disease controls (metabolic/mitochondrial myopathies, neurogenic myopathies including amyotrophic lateral sclerosis and polyneuropathy, and myositis including inclusion body myositis and dermatomyositis) and controls without myopathic changes were included. For details, see eMethods 2.

### Other Analyses

If available, (video) polysomnography (PSG) and electromyography (EMG) were evaluated by a sleep disorders specialist and clinical neurophysiologist (ML). If available, laboratory results (creatine kinase [CK], aspartate transaminase [AST], alanine transaminase [ALT]), brain MRI, and tests on cardiac and pulmonary function were investigated. In serum of patients with proximal muscle weakness, myositis-specific (MSA) and myositis-associated (MAA) antibodies (anti-OJ, -EJ, -PL12, -PL7, -SRP, -Jo1, -PM-Scl75, -PM-Scl100, -Ku, -SAE1, -NXP2, -MDA5, -TIF-1gamma, -Mi-2beta, and -Mi-2alpha) were determined using a lineblot (Euroimmun, Lübeck, Germany). Human leukocyte antigen (HLA) class I and class II genotyping was performed as described.^9^

### Standard Protocol Approvals, Registrations, and Patient Consents

The study was waived by the institutional review board of the Erasmus MC, Rotterdam. Written informed consent was obtained from all patients. The neuropathologic study was approved by the ethics committee of the Medical University of Vienna (EK. Nr.: 1123/2015 and 1636/2019).

### Statistical Analyses

Statistical analyses were performed using IBM SPSS 28.0 (SPSS Inc) for Windows and Prism 8.4.2 (GraphPad). Fisher exact and Fisher-Freeman-Halton tests were used when applicable, while for ordinal data Somers delta was used. Nonparametric tests were used when appropriate.

### Data Availability

Any data not published within this article are available at Erasmus MC, Rotterdam, and Medical University of Vienna. Data will be shared on reasonable request from any qualified investigator, ascertaining anonymization of the individual patients, in line with European privacy regulations.

## Results

Twenty patients with anti-IgLON5 disease were diagnosed in the Netherlands between September 2016 and April 2023. Baseline characteristics are presented in Table 1. Ten patients (50%) were male. The median age at symptom onset was 61.5 years. Nineteen patients (95%) had a chronic disease presentation, in whom symptoms evolved over months to years. One patient with limbic encephalitis had a subacute disease presentation. The median time from symptom onset to diagnosis was 30 months (range 3–280), while 3 already deceased patients were diagnosed retrospectively using stored biofluids.^10^ Subacute deterioration or development of new symptoms was the reason for antibody testing in 12/17 patients (71%). A median of 3 medical specialists were involved before the diagnosis was made. Nine of 20 patients (45%) were first referred to a neurologist specialized in movement disorders. In 4 patients, amyotrophic lateral sclerosis (ALS) was first suspected, because of bulbar symptoms (n = 2) or fasciculations (n = 2). At the time of diagnosis, the median mRS (modified Rankin Scale) was 2 (range 1–4).

**Table 1:** Characteristics of Patients Diagnosed With Anti-IgLON5 Disease

Baseline data	
No. of patients	20
Sex, male (%)	10 (50%)
Age at symptom onset (y, median)	61.5 (IQR 56–71.25, range 45–85)
Time symptom onset to diagnosis, if alive at diagnosis (mo, median)	30 (IQR 21–75, range 3–280)
Number of specialists involved before diagnosis (median)^a^	3 (IQR 2–4, range 2–4)
mRS at diagnosis, if alive (median)^b^	2 (IQR 2–3, range 1–4)
CASE score at diagnosis, if alive (median)^b^	4 (IQR 3–5, range 1–11)
HLA genotyping	
HLA-DRB1*1001 and DQB1*0501	4/16 (25%)
HLA-DQB1*0501	7/16 (44%)
No HLA-DRB1*1001 or DQB1*0501	5/16 (31%)

Abbreviations: CASE = Clinical Assessment Scale in Autoimmune Encephalitis; HLA = human leukocyte antigen; IQR = interquartile range; mRS = modified Rankin Scale.

a In cases 1, 2, and 3, the diagnosis was made after screening a cohort of patients with cognitive symptoms for the presence of autoantibodies using immunohistochemistry; in these cases a neurologist of Erasmus Medical Centre was involved. In cases 4, 6, 7, 9, 11, 12, 13, 15, 17, 19, and 20, the diagnosis was made after phone consultation the treating physician with a neurologist of Erasmus Medical Centre. In both situations this neurologist is included in the number of specialists involved.

b In cases 1, 2, and 3, the diagnosis was made postmortem.

**Figure 1:**
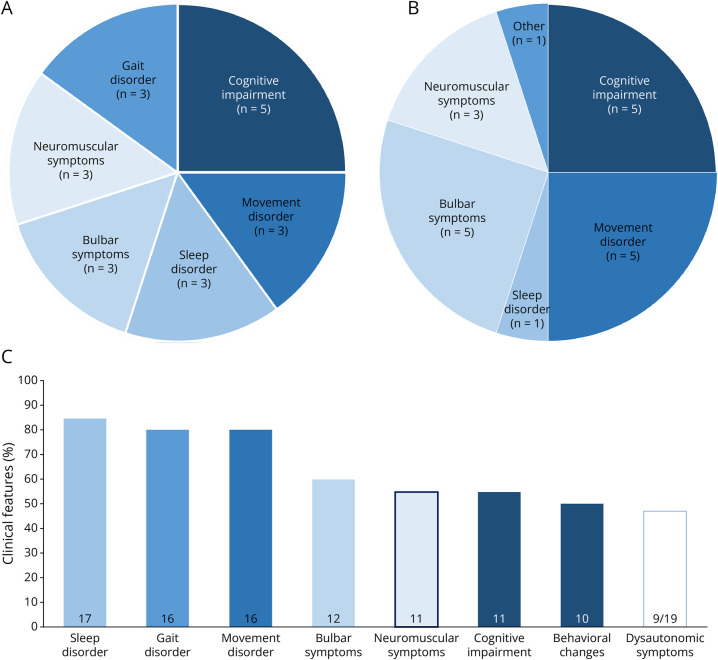
Overview of Clinical Features During Disease Course (n = 20) (A) Initial symptoms. (B) Predominant symptoms at diagnosis. (C) Overview of all clinical features accumulated over the disease course. ^a^Other symptoms consisted of vertigo, malaise, and gait disorder (case 18).

Initial symptoms mainly consisted of cognitive impairment (n = 5, 25%), while movement disorders, sleep disorders, bulbar symptoms, neuromuscular symptoms, and gait disorders evenly occurred in 3 patients (15%). At the time of diagnosis, cognitive impairment, movement disorders, and bulbar symptoms were still prominent (all n = 5, 25%), neuromuscular symptoms were still predominant in 3 patients (15%), but sleep disorders were no longer predominant (n = 1) (Figure 1, A and B).

An overview of the clinical features during the disease course is shown in Figure 1C. Most remarkable, owing to their unexpectedly high frequency, were the neuromuscular signs and symptoms, present in 11/20 patients (55%).

### Neuromuscular Symptoms: Proximal Weakness

Male patients more often had myopathy compared with female patients (8/10 vs 3/10; *p* = 0.07).

In all 11 patients with neuromuscular signs and symptoms, proximal muscle weakness was the clinical sign suggestive of a myopathy (Table 2 and 3). In all of them, both arms and legs were affected, which was symmetrical in 9/11 patients and slightly asymmetrical in 2/11. In all patients, the weakness severity was scored as MRC 4/5.^11^ Six patients (55%) had muscle atrophy. Five patients (45%) had fasciculations, which were generalized in 3 patients, segmental in one patient, and bulbar in one patient. In the patients with generalized fasciculations, these were observed in the atrophic muscles, in the other 2 patients these muscles were not atrophic. Two patients had pain in the affected muscles. Brisk responses of deep tendon reflexes, as can be seen in atrophic muscles in motor neuron disease, were found in 4/6 patients with atrophy. The neuromuscular symptoms did not fluctuate. The maximum walking distance of the patients varied between a few meters and unlimited walking distance (more than 5 km). Two patients used a walker, the other patients did not use any walking aid.

**Table 2:** Clinical Neuromuscular Signs and Ancillary Results in 11 Patients With Clinical Signs of Myopathy

Clinical neuromuscular signs	11/20 (55%)
Proximal weakness^a^	11/11 (100%)
Similar in arms and legs	10/11 (91%)
Symmetric	9/11 (82%)
Atrophy^b^	6/11 (55%)
Generalized	3/6 (50%)
Arms	2/6 (33%)
Legs	1/6 (17%)
Fasciculations^c^	5/11 (45%)
Generalized	3/5 (60%)
Segmental	1/5 (20%)
Bulbar	1/5 (20%)
Ancillary results	
Electromyography	7/11 (64%)
Axonal polyneuropathy^d^	2/7 (29%)
Fasciculations legs > arms^e^	1/7 (14%)
Deviant, nonspecific^f^	2/7 (29%)
Normal	2/7 (29%)
Laboratory tests	
Normal CK	9/9 (100%)
Normal AST or ALT	11/11 (100%)
Normal myositis blot^g^	11/11 (100%)
Cardiac function^h^	2/11 (18%)
Normal	2/2 (100%)
Lung function	4/11 (55%)
Normal	4/4 (100%)

Abbreviations: ALT = alanine transaminase; AST = aspartate transaminase; CK = creatine kinase.

a The legs and arms were equally affected in all but one patient, in whom the legs were more affected. Proximal weakness was slightly asymmetrical in 2/11. In one of them, proximal weakness in the arms was symmetrical, but asymmetrical in the legs.

b Three patients had generalized atrophy (in one patient the arms were more affected than the legs), and in 3 patients, either the muscles of the arms or the legs were atrophic (n = 1 upper arms, n = 1 both upper and forearms, n = 1 thighs).

c Fasciculations were generalized in 3 patients (in one patient, the arms were more affected than the legs), segmental (in lower legs) in one patient, and bulbar in one patient. The severity of the fasciculations was moderate in the patients with generalized fasciculations and mild in the patients with segmental and bulbar fasciculations.

d In 2 patients without fasciculations, asymptomatic axonal polyneuropathy was found during examination. Both patients had loss of vibration sense of both legs and hyporeflexia. In one of them, excessive alcohol use as a risk factor for polyneuropathy was present.

e Fasciculations found in 3/6 tested muscles; in dorsal interosseous muscles on the right side and tibialis anterior on the left side. This can be a sign of peripheral hyperexcitability or motor neuron disease.

f The nonspecific changes consisted of diffuse polyphasic motor unit potentials in case 14, which can be seen in myopathic or neurogenic conditions, and neuropathy in case 17, which was considered to be unrelated to anti-IgLON5 disease.

g A myositis blot testing for antibodies against antigens associated with myositis was performed in serum, this included anti-OJ, -EJ, -PL12, -PL7, -SRP, -Jo1, -PM-Scl75, -PM-Scl100, -Ku, -SAE1, -NXP2, -MDA5, -TIF-1gamma, -Mi-2beta, and -Mi-2alpha. In one patient (case 14), anti-PM-Scl75 was weak positive, while anti-PM-Scl100 was negative, which is an aspecific result.

h Two other patients had a normal ECG (ECG) without symptoms of heart disease. In 7 patients cardiac function was unknown, one of them had a medical history of atrial fibrillation, the other patients had no symptoms of heart disease. None of the patients had clear signs of cardiomyopathy.

**Table 3:** Clinical Neuromuscular Signs and Muscle Biopsy Findings

Case ID, sex, age onset disease (y)	Age onset myopathy (y), time myopathy onset to biopsy (m)	Neuromuscular features	Muscle biopsy findings					
			Myopathic	Immune cell infiltrates/HLA upregulation	Neuro-genic	Ultra-structure	IHC IgLON5	WB IgLON5
Cohort A^a^								
4, M, 45-49	65–69, 8	2020 fasciculations; 2021 progressive prox. limb weakness, atrophy m. deltoideus, DTR brisk	COX neg, RRL fibers, type I predominance, mild type II fiber atrophy, nuclear clumps	No	No	n.a.	Strong SL and mild CM	n.a.
5, M, 55-59	65–69, 13	Prox. axial and limb weakness, progressive despite stop of prednisone, generalized muscle atrophy, DTR decreased	Atrophic fibers, nuclear clumps, type I fiber predominance	Scattered CD8^+^ T cells	No	Crene-lated SL	CP	n.a.
6, F, 55–59^b^	70–74, —	Minimally progressive prox. limb weakness, DTR decreased	n.a.	n.a.	n.a.	n.a.	n.a.	n.a.
10, M, 60-64	60–64, 26	2017 fasciculations; 2019 atrophy limbs/back; 2021 prox. limb weakness, DTR normal	Atrophic fibers, nuclear clumps	No	n.a. (too small)	Mildly crene-lated SL	CP	n.a.
11, M, 60-64	65–69, 4	2020 proximal limb weakness, DTR normal, except for absent Achilles reflex	Type II fiber atrophy, type I predominance, nuclear clumps	No	No	n.a.	n.a. (no tissue left)	n.a.
13, F, 75-79	75–79, 7	2020 falls; 2021 prox. weakness, mild distal weakness, fasciculations, atrophy in arms, DTR very brisk, abnormal plantar responses	Nuclear clumps, caliber variation, type I predominance; internalized nuclei, rimmed vacuoles	No	No	Crene-lated SL	SC and CP	n.a.
14, M, 45-49	55–59, 104	2013 atrophy upper body and fasciculations; 2018 generalized atrophy and fasciculations; 2022 prox limb weakness, DTR arms very brisk, legs normal	Type II fiber atrophy, type I fiber predominance, nuclear clumps	No	Yes^d^	Crene-lated SL	SC and CP	n.a.
17, M, 45-49	45–49, 13	2022 myalgia, progressive prox. limb weakness, limb atrophy, fasciculations, DTR legs very brisk, arms normal	Only paraffin-embedded tissue available; mild fiber atrophy	No	n.a.	n.a.	n.a.	n.a.
18, M, 60-64	60–64, 16	2022 progressive gait disorder, prox. weakness, DTR normal	Type II fiber atrophy, internalized nuclei	No	No	n.a.	n.a.	n.a.
19, M, 70-74	70–74, 37	2019 unsteady gait, no neurologic examination; 2022 prox. limb weakness, myalgia, DTR decreased	Type II fiber atrophy, internalized nuclei, nuclear clumps, COX neg, RRL fibers	No	Yes^d^	n.a.	n.a.	n.a.
20, F, 75-79	75–79, —	2021 gait disorder; 2023 myalgia, prox. limb weakness, dropped head, DTR normal	n.a.	n.a.	n.a.	n.a.	n.a.	n.a.
Cohort B^c^								
1, F, 50–54	65–69, 0,5	2015 facies myopathica with ptosis, prox. weakness	Type II fiber atrophy, nuclear clumps	HLA class I upregulation	No	Crene-lated SL	SL and CP	**+**
2, M, 80-84	Post–mortem	No clear myopathic features, post-mortem muscle biopsy	Type II fiber atrophy, nuclear clumps, single atrophic fibers	Scattered CD3/CD8^+^ T cells in ED	No	n.a.	Predominant CP	**++**
3, F, 50–54	50–54, 4	2017 no clear myopathic features (only stable symptoms of polyneuropathy)	Caliber variation	Mild CD4/CD8^+^ ED	Yes	Unre-mar-kable	Predominant CP	n.a.

Abbreviations: CD = cluster of differentiation; COX = cytochrome oxidase; CP = cytoplasm; DTR = deep tendon reflexes; ED = endomysium; F = female; HLA = human leukocyte antigen; IHC = immunohistochemistry; L = left; M = male; n.a. = not available; PNP = polyneuropathy; prox. = proximal; R = right; RRL = ragged-red like; SL = sarcolemma; WB = Western blot.

a Cohort A is the Dutch cohort of patients with anti-IgLON5 disease.

b In cases 6 and 20, no muscle biopsy was performed.

c Cohort B is the cohort of non-Dutch patients with anti-IgLON5 disease of whom muscle biopsies had been sent to Vienna as part of regular care or second opinion.

d Target fibers, mild fiber type grouping.

In 3 patients (cases 10, 11, and 17; 27%), proximal weakness was the first manifestation of the disease, while non-neuromuscular symptoms developed later in the disease course. Eight of 11 patients (73%) were first referred to a neuromuscular or movement disorders specialist (the latter because of gait difficulties).

Six of 11 patients with neuromuscular complaints had physiotherapy to improve muscle strength and overall condition. Interestingly, 3 patients spontaneously mentioned that physiotherapy increased muscle fatigue and fasciculations, and they therefore had to quit the training.

### Ancillary Testing

#### Muscle Biopsy

A muscle biopsy of the vastus lateralis muscle was performed in 9/11 patients with clinical signs of myopathy (cohort A). Two patients refused muscle biopsy. The biopsy was performed during diagnosis workup before immunotherapy, except for one patient (case 5), who had progressive neuromuscular symptoms and had been treated with oral steroids, IV immunoglobulins (IVIg) and rituximab, but all had been stopped more than 6 months before biopsy.

In addition, 3 muscle biopsies and 1 sural nerve biopsy of non-Dutch patients with anti-IgLON5 disease (of which one deceased patient in whom the biopsy was obtained at autopsy) sent to Vienna for regular care or second opinion were analyzed in parallel (cohort B).

In all 12 muscle biopsy specimens, one or more of the following abnormalities were found (Table 3).

##### Myopathic Changes

**Figure 2:**
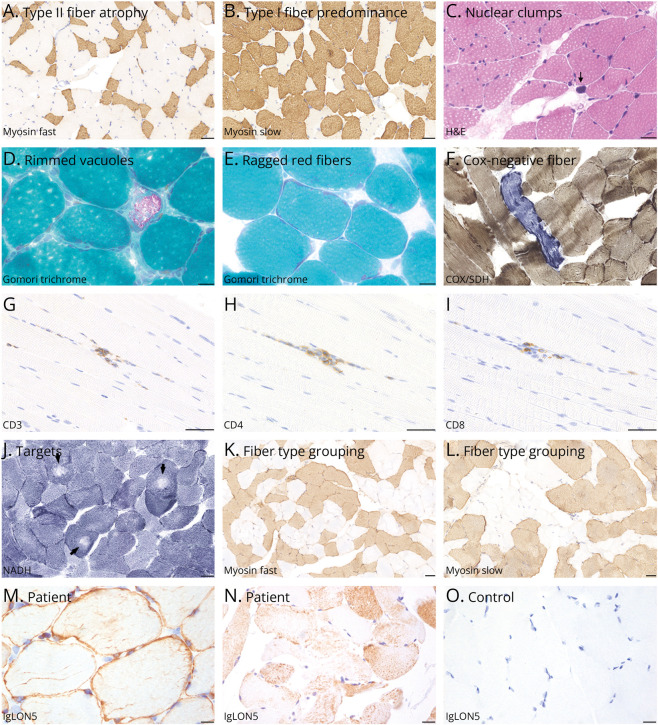
Muscle Biopsy Findings in Patients With Anti-IgLON5 Disease Muscle biopsies of anti-IgLON5 disease patients revealed myopathic changes (A–F), mild inflammatory changes (G–I), and features of neurogenic lesion (J-L). Myopathic changes included fiber type II atrophy (A), fiber type I predominance (B), nuclear clumps (C, arrow), rimmed vacuoles (D), ragged red fibers (E), and COX-negative fibers (F). Mild immune cell infiltration was characterized by endomysial CD4^+^ and CD8^+^ T cells (G–H); in addition, one patient showed upregulation of HLA-class I molecule (I). Three patients showed neurogenic muscle lesions with signs of denervation—target fibers (J, arrows), and reinnervation with fiber type grouping (K; fast myosin; L; slow myosin). Immunoreactivity for IgLON5 showed a strong membrane and cytoplasmic staining pattern (M) or predominantly cytoplasmic staining (N), whereas a healthy control muscle was negative (O). Images A–C and I are from patient 1, cohort B; Image D is from patient 13, cohort A; Images E–F and M are from patient 4, cohort A; Images G–I are from patient 3, cohort B; Images J–L are from patient 14, cohort A; Image N is from patient 5, cohort A; Scale bars: A, B; F–H, J–L: 60 μm; C–E; I; M–O: 30 μm. IgLON5 = immunoglobulin-like cell adhesion molecule 5.

In all patients, muscle biopsies showed mild myopathic alterations, including single atrophic fibers (n = 12), type II fiber atrophy (n = 7, Figure 2A), type I fiber predominance (n = 5, Figure 2B), nuclear clumps (n = 9, Figure 2C), internalized nuclei (n = 3), rimmed vacuoles (n = 1, Figure 2D), or ragged red-like fibers including COX-negative fibers (n = 2, Figure 2, E and F). None of the muscle biopsies showed deposition of phosphorylated tau, TDP43, p62, or ubiquitin (data not shown).

##### Immune Cell Infiltration

We found mild immune cell infiltration in 3 muscle biopsies. In the first case (cohort A, case 5), biopsy of the vastus lateralis muscle showed mild unspecific alterations with some isolated atrophic fibers and clumps of nuclei. Immunohistochemistry revealed scattered CD8^+^ and CD4^+^ T cells in the endomysium. However, CD20^+^ and CD79a+ B cells as well as complement C5b-9 were negative.

In the second case (cohort B, case 2), biopsy of the vastus lateralis muscle showed nonspecific myopathic changes with nuclear clumps, pronounced atrophy of type 2 fibers and mild infiltration of CD8^+^ and CD4^+^ T cells in the endomysium. Again, CD20^+^ and CD79a+ B cells were absent.

In the third case (cohort B, case 3), sample of the gastrocnemius muscle showed a chronic neurogenic atrophy with groups of angulated fibers and fiber type grouping. In addition, some CD4^+^ and CD8^+^ T cells (Figure 2, G and H) and CD68^+^ macrophages were visible in the endomysium, compatible with a mild interstitial myositis. Again, immunohistochemistry for CD20, CD79a, and complement deposition (C5b-9) was negative. A sample of the sural nerve showed a moderate chronic axonal neuropathy with loss of approximately 40% of large myelinated nerve fibers in all fascicles and increase in endoneurial connective tissue along with axonal regeneration as indicated by the presence of regenerating clusters. In addition, a demyelinating and remyelinating component was visible. Immunohistochemistry revealed some CD8^+^ T cells in the endoneurium.

One case (cohort B, case 1) showed an upregulation of HLA class I molecules on some fibers (Figure 2I) but no immune cell infiltration. None of the 9 IgLON5 biopsies showed T-cell invasion into intact muscle fibers or deposition of terminal complement complex C5b-9 on the sarcolemma or capillaries. No IgG or IgG4 deposition was found on the sarcolemma (investigated in 8 muscles with enough tissue left).

##### Neurogenic Lesion

A neurogenic lesion was found in 3 cases. One case was combined with mild inflammation (see above cohort B, case 3). In the other 2 cases (cohort A, cases 14 and 19), muscle biopsies were performed 8 and 3 years after onset of myopathy symptoms, respectively. Histologic examination in both biopsies showed prominent target fibers (Figure 2J) and mild fiber type grouping (Figure 2, K and L) but no groups of atrophic fibers.

##### IgLON5 Expression

We investigated the expression of IgLON5 in muscles by immunohistochemistry for IgLON5, performed on frozen or formalin-fixed and paraffin embedded muscle (investigated in 8 muscle biopsies with enough tissue left). We observed either a strong immunoreactivity at the sarcolemma along with cytoplasmic staining in 4 cases (Figure 2M), or a predominantly cytoplasmic staining in 4 cases (Figure 2N). In 4 healthy control muscles, IgLON5 immunoreactivity at the sarcolemma and in the cytoplasm was either faint or absent (Figure 2O). By contrast, it was strongly upregulated in atrophic and regenerating fibers in neurogenic muscle lesion (n = 1), metabolic/mitochondrial myopathy (n = 3), and dermatomyositis (n = 1, data not shown).

**Figure 3:**
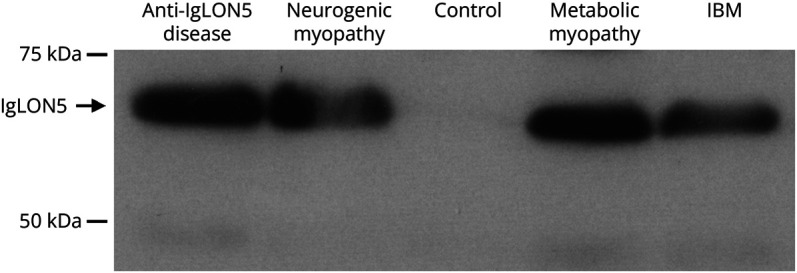
Western Blot Analysis Anti-IgLON5 immunoblot shows high IgLON5 levels in a muscle specimen from an anti-IgLON5 disease patient (first lane: postmortem muscle tissue of case 2, cohort B) as well as in muscle tissue from patients with neurogenic atrophy (second lane: postmortem muscle tissue of a patient with amyotrophic lateral sclerosis), mitochondrial myopathy (fourth lane: muscle biopsy), and inclusion body myositis (fifth lane: muscle biopsy). By contrast, IgLON5 is absent in a healthy control muscle (third lane: muscle biopsy). IgLON5 = immunoglobulin-like cell adhesion molecule 5.

In line with the pronounced IgLON5 immunoreactivity on immunohistochemistry, we detected increased protein levels of IgLON5 using Western blotting of total protein extracts from muscle biopsy and autopsy samples from 2 anti-IgLON5 disease patients, 4 cases with neurogenic myopathy, 2 metabolic/mitochondrial myopathy cases, and 2 patients with inclusion body myositis (Figure 3). IgLON5 expression was very weak or absent in 2 healthy control muscles, indicating a feature observed in myopathies of different origin, not restricted to anti-IgLON5 disease.

#### Ultrastructural Findings

**Figure 4:**
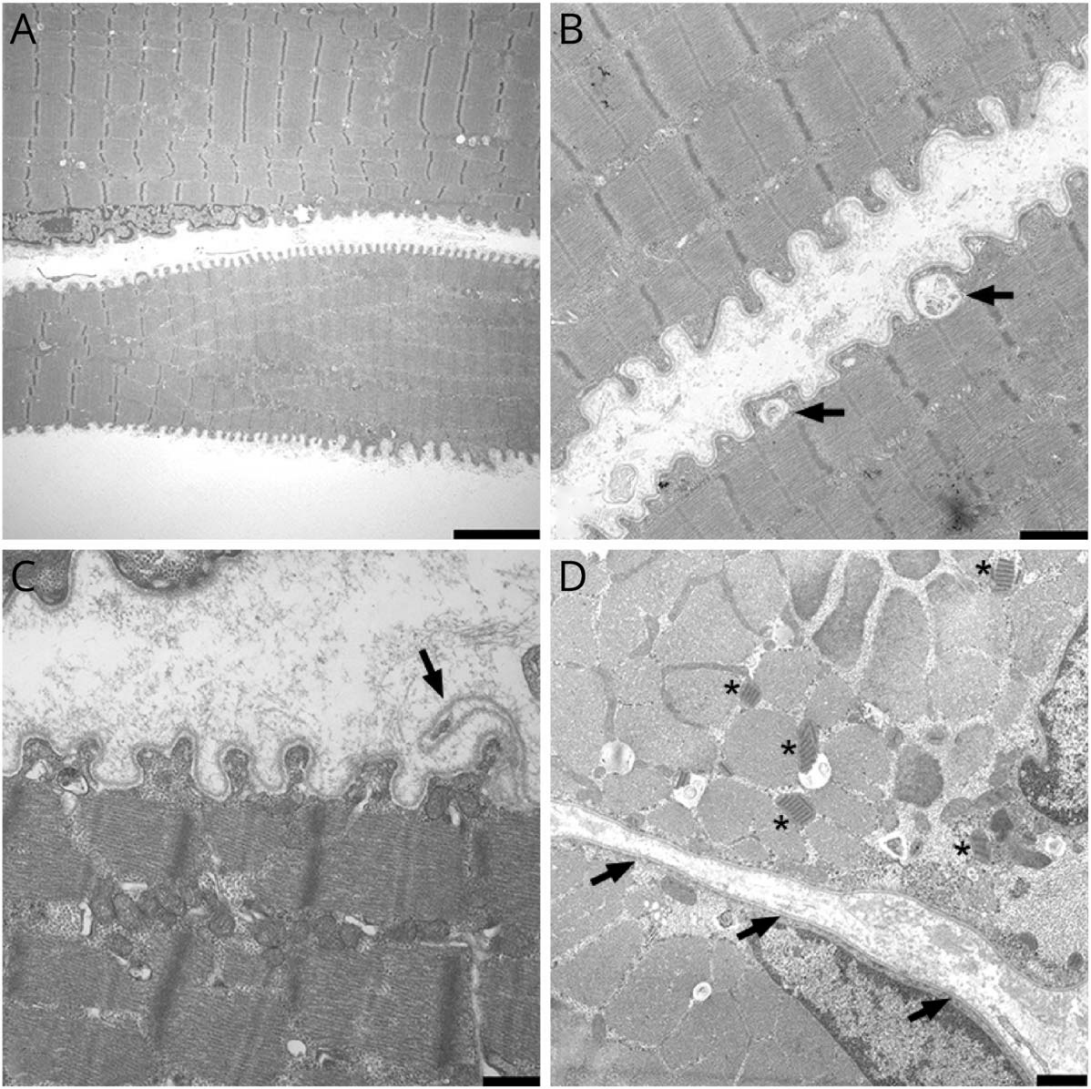
Electron Microscopy Findings in Biopsies of Patients With Anti-IgLON5 Disease Electron microcopy revealed prominent folding of the sarcolemma that affects the entire fiber circumference (A–C). Some of the folds contain vesicles (B, arrows), and all are covered by basal lamina (B–C). A loop of basal lamina surrounds an empty space (C, arrow). Myofibrils and mitochondria are unremarkable. Muscle fibers of a patient with metabolic myopathy do not show folding of the sarcolemma (D). Images A–B are from patient 1, cohort B; Image C is from patient 5, cohort A; Image D is from a patient with metabolic myopathy (mitochondria with paracrystalline inclusions are marked with an asterisk) with upregulation of IgLON5 on immunohistochemistry and Western blot. Scale bars: A: 3.3 μm; B: 2.5 μm; C, D: 2.2 μm. IgLON5 = immunoglobulin-like cell adhesion molecule 5.

Electron microscopic studies were performed in 6 muscle biopsies. Five cases showed prominent formation of sarcolemmal folds in both normotrophic and atrophic fibers. These changes, best described as crenelations, covered the entire fiber circumference in some (Figure 4A) or only a segment in others. Some of the folds contained endocytic vesicles. They were always covered by a basal lamina (Figure 4B, arrows). Occasionally, loops of basal lamina surrounded empty spaces, suggesting disappearance of preexisting folds (Figure 4C, arrow). The myofibrils and mitochondria were unremarkable. Crenelation of the sarcolemma was absent in 2 muscle biopsies with metabolic/mitochondrial myopathy (Figure 4D).

Limited due to the small sample size, no obvious relationship of myopathic or other specific biopsy features with duration of myopathy, HLA genotyping, anti-IgLON5 IgG subclasses, or antibody titers was observed.

#### Electromyography

In the Dutch cohort A, EMG was performed in 7/11 patients with proximal muscle weakness. In none of the patients specific myopathic alterations—for example, myopathic motor unit potentials (MUP)—were found, while less specific findings such as fibrillations, positive sharp waves, or complex repetitive discharges were identified in several patients (Table 2).

In 4/5 patients with clinical fasciculations, EMG was performed. It showed electrographic fasciculations in arms and legs, without fulfilling the El Escorial criteria or the Gold Coast criteria for the latter,^12,13^ and in another patient with generalized fasciculations an axonal neuropathy was found. Three patients in whom a sufficient number of muscles were tested, 2 with clinical fasciculations, did not fulfill the El Escorial criteria for motor neuron disease.^12^

#### Laboratory Tests

None of the 11 patients had laboratory signs of muscle damage. Levels of CK, ALT, and AST were normal. Furthermore, no MSA and MAA antibodies could be detected in these patients.

IgLON5-specific IgG subclasses were measured in pretreatment serum samples using CBA. No relation between anti-IgLON5 IgG subclasses and myopathy was found.

#### Cardiac and Lung Function Tests

None of the patients had clear signs of cardiomyopathy. Lung function tests were performed in 4 patients and were all normal.

## Discussion

This study identified proximal myopathy as a prominent clinical feature in anti-IgLON5 disease, which might be related to a functional impairment of IgLON5 in muscle regeneration. This finding broadens the clinical phenotype of anti-IgLON5 disease, provides insight in underlying pathophysiologic mechanisms, and can be an important clue for earlier diagnosis and start of immunotherapy.

In our cohort, over half of the patients had proximal muscle weakness, mostly in combination with atrophy or fasciculations. To date, there have been some reports about neuromuscular symptoms in anti-IgLON5 disease. In 2017, Wenninger^14^ reported the first such patient, a 58-year-old man with fasciculations, progressive proximal muscle weakness, and mild muscle wasting. Afterward, neuromuscular symptoms were mentioned in several studies, including proximal or distal weakness, bulbar symptoms, atrophy, fasciculations, cramps, or spasticity.^1,4,6,15-20^ The neuromuscular symptoms were mostly mentioned as a less relevant symptom in a minority of the patients. The timing of appearance of neuromuscular symptoms during the disease course or a possible relation with treatment, i.e., corticosteroids, was mostly not described. A link with anti-IgLON5 disease was not extensively investigated. In our cohort, neuromuscular symptoms were seen remarkably frequent and therefore became the focus of our study, although symptoms could be relatively mild. The clinical phenotype of proximal muscle weakness and atrophy is most suggestive of myopathy. However, fasciculations are not often seen in myopathy and could be explained by peripheral nerve hyperexcitability or motor neuron localization. Using EMG, none of the patients in whom sufficient number of muscles were tested, fulfilled the El Escorial criteria for motor neuron disease.^12^ Myopathic changes, for example, myopathic motor unit potentials, were not found by EMG either. However, differentiating between a myopathic and neurogenic cause with EMG is often not possible, since over time pathologic motor unit potentials in myopathy will take on a more neurogenic appearance.^21^ Two of our muscle biopsies showed signs of neurogenic atrophy with target fibers and fiber type grouping, which can also point towards motor neuron disease or peripheral neuropathy. Taken together, the clinical phenotype, EMG, and muscle biopsies showing myopathic changes along with upregulation of IgLON5 mostly point toward a pathology in the muscles, either isolated or complemented by peripheral nerve or motor neuron involvement. This hypothesis is further supported by the intriguing finding reported by some of our patients that physiotherapy seemed to worsen symptoms, which might be attributed to IgLON5 antibodies hampering normal physiologic regenerative capacities after different kinds of damage (e.g., systemic, neurogenic). To the best of our knowledge, in none of the previously reported anti-IgLON5 disease patients, neuromuscular symptoms were investigated by muscle biopsy. Thus, our study supports that myopathy at least in part underlies the neuromuscular symptoms in anti-IgLON5 disease.

In all patients with proximal muscle weakness, biopsy of the vastus lateralis muscle showed mild myopathic alterations. In addition, in some patients mild T-cell infiltration was found, while in others, neurogenic changes were seen. Morphological features were globally unspecific, as type 2 fiber atrophy may be detected in several conditions and may be a secondary effect of reduced muscular activity and corticosteroids, single COX negative, or ragged red fibers may be detected in elderly population, and a crenelated sarcolemma has been described also in other conditions.^22,23^ However, we found a strong upregulation of IgLON5 in muscle fibers in all examined anti-IgLON5 patients, which was equal to that observed in different muscle diseases including neurogenic, metabolic/mitochondrial, or inflammatory myopathy, while IgLON5 expression in healthy control muscle was weak or absent.

IgLON5 is a cell-adhesion molecule of unknown function, which is attached to the cell membrane by a glycosylphosphatidylinositol-anchor.^24^ A proteomic screen of neuronal cell surface molecules revealed that IgLON5 interacts as homodimer and heterodimer and suggested a role in interaction across the synaptic cleft.^25^ A subsequent study demonstrated that antibodies from patients with anti-IgLON5 disease prevent the protein interaction of soluble IgLON5 with other IgLON family members in transfected HEK cells and in rat hippocampal neurons.^26^ Recently, studies on C2C12 myoblasts revealed that IgLON5 also plays a role in myogenesis and muscle regeneration by regulating the adhesion and differentiation of myoblasts.^27^ Similarly, IgLON5 might play a role in regeneration of muscle fibers in a variety of pathologic processes in humans. In our study, we found a paradoxical upregulation of IgLON5 protein expression in anti-IgLON5 disease, which is analogous to that observed in various muscle diseases including denervation, metabolic, or inflammatory myopathy and most likely reflects a response to muscle damage and regeneration.

Interestingly, although the IgLON5 protein was strongly expressed on the sarcolemma and in the cytoplasm, we could neither detect deposition of whole IgG or IgG4 on the muscular membrane nor deposition of terminal complement complex C5b-9. Moreover, we did not find alterations of myofibrils or deposition of proteins such as Tau or TDP43, which makes it difficult to postulate a direct pathogenic role of anti-IgLON5 antibodies in myopathy of patients. However, we found mild immune cell infiltration in 3 and upregulation of HLA class I antigen in one biopsy specimen. Moreover, ultrastructural investigation revealed abnormal muscle fibers with prominent folds into the extracellular space along the fiber circumference with occasional loops of basal lamina around empty spaces. Similar ultrastructural alterations of the surface membrane have been described in different myopathies including different forms of muscular dystrophies or dermatomyositis and were discussed to either arise from fiber atrophy, defects of muscle membrane stability, extrusion of cytoplasmic degradation products by exocytosis, or sculpturing of the membrane by cytotoxic lymphocytes.^22,23^ Since IgLON5 is an adhesion molecule that establishes homomeric and heteromeric interactions with other IgLON family members and undergoes spontaneous ectodomain shedding,^26^ it could also be hypothesized that the sarcolemmal folds may result from functional blocking of IgLON5 interactions. Further investigations on muscle cell lines will be necessary to decipher potential effects of anti-IgLON5 antibodies on muscular regeneration and sarcolemmal folding.

In our cohort, we did not find a relationship between myopathic features and time from myopathy onset to biopsy, possibly due to the low sample size, as we had expected to see inflammation first, followed by degeneration. We did not perform MRI of proximal muscles to identify potential inflammatory changes to guide the biopsy. This could have increased the yield to detect inflammatory abnormalities.

Our study has several limitations. First, our Dutch cohort consisted of only 20 patients, and myopathy diagnostics could be performed in even less patients. The power to detect relevant associations, even those expected based on theories, was therefore low. Second, part of the information has been retrospectively collected. In addition, our cohort, despite being nationwide, bears a selection bias: antibody testing in the Netherlands is more frequently considered in some subspecialities such as movement disorders or neuromuscular disorders and is less frequently requested in sleep disorders. This can differ in other countries, resulting in different research groups seeing their own tip of the iceberg. This makes simple comparisons between different cohorts more complicated. Recently, Gaig et al.^28^ developed the anti-IgLON5 disease composite score (ICS) to assess the extension and severity of symptoms in anti-IgLON5 disease, using 17 symptoms divided into 5 clinical domains. However, neuromuscular symptoms are underrepresented in this score, since only fasciculations are part of it. Addition of other neuromuscular symptoms, such as proximal weakness or atrophy, should be considered.

Apart from these limitations, our study has several strengths. Detailed information was gathered on all patients. We assessed all patients (alive at time of diagnosis) ourselves, providing us with the opportunity to perform extensive interviews and assess all aspects of the disease, including a thorough consideration of the neuromuscular symptoms. Myopathy as part of the clinical spectrum of anti-IgLON5 disease could be confirmed using structured muscle biopsy analysis and excluding iatrogenic causes. Although availability of muscle biopsy tissue was relevant to research and understanding, muscle biopsies are not necessary to establish the diagnosis of anti-IgLON5 disease and are probably not essential in routine care.

To conclude, we found proximal myopathy as a prominent clinical feature in anti-IgLON5 disease, which might result from impaired function of IgLON5 in muscle regeneration. These findings broaden the clinical phenotype of anti-IgLON5 disease and can represent an important clue for earlier diagnosis and start of immunotherapy. In all patients with the neuromuscular symptoms here described (proximal weakness, atrophy, or fasciculations) or atypical ALS-like symptoms, the possibility of anti-IgLON5 disease should be considered.

## Glossary

ALS: amyotrophic lateral sclerosis
ALT: alanine transaminase
AST: aspartate transaminase
ATP: ase adenosine triphosphatase
CBA: cell-based assay
CD: cluster of differentiation
CHO: Chines hamster overian
CK: creatine Kinase
COX: cytochrome oxidase
EMG: electromyography
H&E: hematoxylin and eosin
HLA: human leukocyte antigen
IgG: immunoglobulin G
IgLON5: immunoglobulin-like cell adhesion molecule 5
IHC: immunohistochemistry
IVIg: IV immunoglobulins
MRC: Medical Research Council
mRS: modified Rankin Scale
NADH: nicotinamide adenine dinucleotide
PAS: periodic acid‐Schiff
PSG: polysomnography
SDH: succinate dehydrogenase
